# Implementation and evaluation of community-based drop-in centres for breastfeeding support in Victoria, Australia

**DOI:** 10.1186/s13006-017-0136-7

**Published:** 2017-11-13

**Authors:** Rhian L. Cramer, Helen L. McLachlan, Touran Shafiei, Lisa H. Amir, Meabh Cullinane, Rhonda Small, Della A. Forster

**Affiliations:** 1 Judith Lumley Centre (formerly Mother and Child Health Research), La Trobe University, 215 Franklin St, Melbourne, VIC 3000 Australia; 2 School of Nursing, Midwifery and Healthcare, Federation University Australia, University Drive, Mount Helen, Ballarat, VIC 3350 Australia; 3 School of Nursing & Midwifery, La Trobe University, Bundoora, VIC 3086 Australia; 4 Department of Women’s and Children’s Health, Karolinska Institutet, Retzius väg 13A, Stockholm, Sweden; 5 The Royal Women’s Hospital, Cnr Grattan St and Flemington Rd, Parkville, VIC 3052 Australia

**Keywords:** Breastfeeding, Breastfeeding support, Breastfeeding promotion, Drop-in centre, Outpatient service, Community health services, primary health care, preventative health care

## Abstract

**Background:**

While Australia has high breastfeeding initiation, there is a sharp decline in the first weeks postpartum and this continues throughout the first year. Supporting breastfeeding In Local Communities (SILC) was a three-arm cluster randomised controlled trial to determine whether early home-based breastfeeding support by a maternal and child health nurse (SILC-MCHN), with or without access to a community-based breastfeeding drop-in centre, increased the proportion of infants receiving *any* breast milk at three, four and six months. The trial was conducted in ten Local Government Areas (LGAs) in Victoria, Australia.

The primary aim of this paper is to describe the three drop-in centres established during the trial; and the profile of women who accessed them. The secondary aim is to explore the views and experiences of the drop-in centre staff, and the challenges faced in establishing and maintaining a breastfeeding drop-in centre in the community.

**Methods:**

Evaluation of the three LGAs with drop-in centres was multifaceted and included observational visits and field notes; data collected from attendance log books from each drop-in centre; a written survey and focus groups with maternal and child health (MCH) nurses who ran the drop-in centres; and semi-structured interviews with MCH coordinators of the participating LGAs.

**Results:**

The three LGAs developed and ran different models of breastfeeding drop-in centres. They reported challenges in finding convenient, accessible locations. Overall, attendance was lower than expected, with an average of only one attendee per session. Two global themes were identified regarding staff views: *implementation challenges*, encompassing finding accessible, available space, recruiting volunteers to provide peer support, and frustration when women did not attend; and *the work of SILC-MCHNs*, including themes of satisfying and rewarding work, juggling roles, and benefits to women, babies and the community.

**Conclusion:**

Providing community-based breastfeeding support was satisfying for the drop-in centre staff but proved difficult to implement, reflected by the lower than anticipated attendances at all of the drop-in centres. Interventions to increase breastfeeding in complex community settings require sufficient time to build partnerships with the existing services and the target population; to understand when and how to offer interventions for optimum benefit.

**Trial registration:**

Australian New Zealand Clinical Trials Registry ACTRN12611000898954.

## Background

Australia has a high rate of breastfeeding initiation, with approximately 96% of women breastfeeding at least once whilst in hospital [1]. However, there is a sharp decline in breastfeeding rates in the first weeks after birth and the decline continues throughout the first year [1, 2]. There is also a widening gap between high and low socioeconomic populations, with infants in poorer social circumstances less likely to receive breast milk [2]. In Victoria, Australia, breastfeeding rates vary widely between Local Government Areas (LGAs), ranging from 29% to 77% of infants receiving *any* breast milk at 6 months [3, 4].

We undertook a trial to introduce and evaluate an intervention aimed at increasing breastfeeding rates by providing community-based breastfeeding support in the early postpartum period [5, 6]. Supporting breastfeeding In Local Communities (SILC), was a three-arm cluster randomised controlled trial (RCT), including ten local government areas (LGAs) in the state of Victoria, with LGAs as the unit of randomisation [5]. LGAs that had a lower than average rate of women providing *any* breast milk to their infants at discharge from hospital, with greater than 450 births per year, and that agreed to participate, were randomly allocated to one of three trial arms: 1) standard care (comparison arm); 2) home-based breastfeeding support (home visits, HV); or 3) home-based breastfeeding support plus access to a community-based breastfeeding drop-in centre (HV plus drop-in centre). Maternal and Child Health Nurses (MCHNs) were recruited by each LGA (called SILC-MCHNs) to provide the home-based breastfeeding support (all SILC-MCHNs) as well as to staff the community-based breastfeeding drop-in centres (only SILC-MCHNs in the HV plus drop-in centre trial arm). Further details of the recruitment and randomisation process can be found in the SILC trial protocol [5].

The intervention was pragmatically designed so that if such an intervention did increase breastfeeding rates then it would be able to be readily incorporated into practice in Victoria. It was important during this trial not only to investigate if the interventions resulted in a clinically important difference in breastfeeding rates at four and six months, but also to test the feasibility of implementing the interventions. The home-based breastfeeding support was designed for women at risk of ceasing breastfeeding before six months and the trial protocol guided LGAs in how to assess women for this visit [5].

Overall the trial found no difference in breastfeeding outcomes between standard MCH care and either of the intervention trial arms [6]. Given the findings, process evaluation measures to ascertain whether the interventions were implemented as planned are crucial in explaining and understanding the trial outcome. Data collection for the process evaluation included measuring adherence to the SILC protocols, measurements of intervention exposure, and exploring the views and experiences of the SILC-MCHNs and the MCH coordinators at each of the six intervention LGAs. A description of the content of the SILC-MCHN home visits has been published separately [7].

The Victorian Government Department of Education and Early Childhood Development (DEECD) commissioned the SILC trial with the intent to design, implement and evaluate innovative breastfeeding interventions to improve breastfeeding rates in the state of Victoria. Drop-in centres offering breastfeeding support within community settings was one innovation strongly supported by MCHNs [8], as it was felt they provided the opportunity to provide not only breastfeeding support, but also social support and education, which is widely discussed in the breastfeeding literature as a key factor in breastfeeding success [9, 10].

There have been few evaluations of breastfeeding drop-in centres published; even less focussed on drop-in centres with multifaceted professional, community-based breastfeeding support. The systematic reviews that reported on community support for breastfeeding were about the provision of peer support [11–13]. Of those papers that did describe and/or evaluate professional breastfeeding support services, the majority were located within or adjacent to hospitals [14–16]. A small number of publications were identified that described informal drop-in centres for breastfeeding mothers to be able to access within their own community [17–20]. Table 1 shows all identified studies of breastfeeding drop-in centres and gives an overview of each study. Breastfeeding drop-in centres were effective in improving women’s satisfaction with their breastfeeding experience [15, 18, 20], and authors reported increased breastfeeding duration [18, 21] to four [15] and/or six months [15, 17]. One evaluation of existing drop-in services compared the breastfeeding rates of drop-in centre attendees to the population data for the local area [15]. Two studies found that women motivated enough to seek breastfeeding support, such as those women seeking support at a drop-in centre, were more likely to be determined to breastfeed and overcome any barriers they encountered [22, 23]. There is strong evidence that motivation to breastfeed is one of the most important predictors of breastfeeding success [22, 23], so comparison to the population data may be misleading and therefore results from these studies need to be interpreted with caution.

**Table 1:** Summary of publications about breastfeeding drop-in centres (alphabetical order, by first author)

First author [reference] Journal, year	Location Drop-in services offered	Methodology; Data collection	Response fraction	Comments
Adams et al. [15] JOGNN, 2001	Dufferin County, Ontario, Canada Hospital-based clinic (2.5 h, 3 times per week)	Retrospective service evaluation	164/242* (68%) *Numbers of respondents reported are inconsistent throughout paper	▪ Drop-in centre located within a hospital, next to the obstetric unit ▪ Access to the drop-in centre was for women seeking breastfeeding support, both as inpatients and after discharge ▪ Participants were surveyed on their experiences and satisfaction with the drop-in centre services ▪ Data collected was compared to the previous data available on breastfeeding women from the local area
Berridge et al. [14] Maternal and Child Nutrition, 2005	North-west England Hospital-based clinic (1 morning per week) + telephone help line	Exploratory, descriptive study; Written questionnaire, Field notes	80/108 (74%)	▪ Drop-in centre run as a clinic within a hospital, in the antenatal parentcraft room ▪ Written questionnaire was the primary data source. However, informal conversations between the researcher and the women attending the drop-in centre allowed in-depth field notes to be collected ▪ Targeted clients with a lower socioeconomic status (SES) but results showed a higher SES sample, with older, more educated participants
Caddy [16] The Practising Midwife, 2002	Reading, England Hospital-based drop-in clinic (2 h, 3 times per week)	Description of services	Not reported	▪ Thorough description of the considerations of running a drop-in service; particularly timing, venue, staffing, advertising and funding ▪ This was a descriptive paper, not an evaluation so there are no data on the efficacy of the services
Fox et al. [19] BMC Pregnancy and Childbirth, 2015	UK Community-based drop-in sessions	Qualitative, descriptive study; 47 interviews (41 in person, 6 phone) 5 focus groups	51/63 (81%)	▪ Drop-in centres run in the community by professionals and peer supporters ▪ Multiple sites included in this review: A range of metropolitan, regional and remote sites ▪ Did not invite women experiencing acute BF problems to participate ▪ Interviews were completed during the drop-in sessions, resulting in interruptions and premature ending of the interview
Pastore & Nelson [17] Journal of Human Lactation, 1997	Richmond, Canada Based in community centre (3 h per week)	Descriptive study; Semi-structured telephone interviews	57/62 (92%)	▪ Community-based drop-in centre staffed by lactation consultants and child health nurses ▪ Women were interviewed via phone on their views and experiences of breastfeeding support from the drop-in centre ▪ Positive feedback from the women using the service but, as this was designed as a descriptive study, there was no ability for evaluation of the effect of the service on breastfeeding rates
Price [20] Community Practitioner, 2014	Berkshire, England Based in community health centre (frequency and duration of sessions not reported)	Service evaluation Written questionnaire	15 (not recorded)	▪ Multifaceted BF support service review (drop-in centre breastfeeding support was only one aspect of the evaluation) ▪ Sought feedback from the women using the centre at the time of their attendance on two days over the 6 month evaluation, therefore has a small sample size ▪ Breastfeeding outcomes of this trial were given as an overall rate for the local area and were not specific to the impact of the drop-in centre
Stefiuk et al. [18] Journal of Human Lactation, 2002	Saskatoon, Canada Community-based clinic (weekdays, with telephone consultation on weekends)	Descriptive process evaluation; Phone interviews, Observation of visits Review activity logs/administrative paperwork, Self-administered written questionnaire	Written questionnaire and follow up phone interview: 43/50 (86%) Phone interview only: 25/30 (83%) Observed visits: 4 (22%)	▪ Multifaceted design ▪ No data presented on numbers of attendances at drop-in centre or how many were contacted via telephone or how they were selected ▪ Not designed to evaluate the effect of the drop-in centre on breastfeeding but reported women’s perceptions that the drop-in centre support helped them to increase their breastfeeding duration

*LC* lactation consultant, *SES* socioeconomic status

Community based drop-in services have been successfully established in many other areas of health and human services. These include services targeting sexual health [24–27]; homelessness [28, 29]; youth services [26, 28, 30–33]; eating disorders [34]; smoking cessation [35, 36]; needle exchange programs and safe injecting rooms [37]. While the services differ greatly in their target group and purpose, the integral components of service provision within community settings are social support [14, 15, 24, 29], community building [30], isolation reduction [14, 29, 30] and referral to other available services within the community [17, 24, 25, 29, 30].

In Victoria, Australia, there are approximately 74,000 births per year [38]. Universal care for mothers and infants is provided by MCHNs, who are skilled clinicians with qualifications in nursing, midwifery, and maternal and child health. Irrespective of a woman’s locality or maternity care provider, once discharged from hospital, every mother is entitled to MCH care, free of charge. A home visit is scheduled within the first two weeks of birth whenever possible, then pre-specified consultations at the local MCH centres are scheduled based on childhood milestones. These visits are known as ‘Key Ages and Stages’ consultations. This support involves ten visits from birth until the child reaches three and a half years of age and the aim is that the health of both mother and child are addressed [39].

The primary aim of this paper is to describe the SILC drop-in centres, how they were established and maintained, and the profile of women who accessed the drop-in services. The secondary aim is to explore the views and experiences of the SILC-MCHNs and MCH coordinators; the perceived benefits of the service; and the challenges faced in establishing and maintaining a breastfeeding drop-in centre in the community.

## Methods

The study used qualitative and quantitative data from surveys, focus groups, interviews, drop-in centre log books and visitors’ comment books, and SILC-MCHN diaries. Each data source is described below. The SILC intervention period was between September 2012 and March 2013, and data for this project were collected between September 2012 and January 2014.

Each LGA was allocated a proportion of the available SILC-MCHN equivalent full time (EFT) funding determined by the number of births (with funding provided by the State government). The three LGAs randomised to having a drop-in centre could allocate these funds to staff the centres to best suit their local community. Each LGA decided on which days and for how many hours each drop-in centre would run, within their allocated funding. Each LGA was given the scope to develop the drop-in services with their own community’s needs in mind, and the location was determined by the LGA. Consequently, each of the LGAs proposed, developed and ultimately ran different styles of drop-in centre. Our intention was for the services to be similar to Baby Cafés in the United Kingdom [19, 40], in that they should be accessible to women; close to public transport; welcoming spaces suitable for new mothers and babies; provide privacy for feeding; and provide access to change tables, toilets and drink facilities. Our aim was that both professional and peer support would be available so that women would be able to discuss breastfeeding issues or concerns with the SILC-MCHN in the centre and at the same time meet other mothers, so that women could learn from each other. LGAs advertised and promoted the drop-in centres within their LGA. A two month run-in period prior to the official trial period facilitated the set-up of the drop-in centres and commencement of service provision in an effort to identify and resolve any challenges encountered. It also gave time to raise community awareness of the drop-in centres.

### Drop-in centre attendance and functioning

Observational visits were conducted at drop-in centres in each of the three LGAs by RLC in March 2013. Informal discussions took place with the SILC-MCHN present regarding opening hours of the drop-in centres, choice of location, and amenities available and detailed field notes were kept. Also discussed was the promotion of the drop-in centres and any advertising conducted to inform women of the drop-in centres available. Photographs of the location and facilities were taken. In the event there were any staff or participants to be included, written consent was obtained prior to the photograph being taken.

During the time the SILC interventions were in place, SILC-MCHNs were asked to maintain tools that helped the research team understand intervention compliance, including log books to record women’s visits to the drop-in centres, visitors’ comment books and SILC-MCHN diaries. Items included in the log book were the date of the visit, infant age, maternal parity, reason for attending the service, and whether this was the first visit to the centre. Diaries were provided for SILC-MCHNs to record their reflections and experiences of running the drop-in centres. They were encouraged to complete one entry per drop-in session to provide a record of their views and experiences of the drop-in centres as they were implemented and became established and then, if applicable, when the service was closed. Visitors’ comment books were provided at each of the drop-in centres and SILC-MCHNs were encouraged to have the women attending the service complete entries on their thoughts and experiences of the drop-in centre.

### Exploring SILC-MCHNs views: Focus group and survey

SILC-MCHNs’ views and experiences of the drop-in centres were sought via a focus group and a short survey. We used the two approaches because we felt some SILC-MCHNs may not be comfortable reporting challenges they may have faced in the focus group context given they knew the other participants, and that the workplaces and LGAs may be identifiable.

The focus group was held in the final month of the intervention (March 2013), at a scheduled SILC-MCHN workshop. SILC-MCHNs could choose to stay on after the workshop and participate in the focus group. The focus group schedule was developed specifically for the study. Topic areas included SILC-MCHNs’ experiences of participating in the SILC trial; the role of the SILC-MCHN; positive aspects of the intervention; and any challenges faced.

Similar topic areas informed the questions in the written survey, which was distributed after the focus group discussion. The survey included demographic characteristics; Likert-type scales exploring SILC-MCHNs’ ability to work autonomously; satisfaction with support received during the trial; confidence in the SILC-MCHN role; perceived sustainability of the drop-in centres; and open-ended questions. The final survey included 26 questions and took 10 to 15 min to complete.

Piloting of the SILC-MCHN survey was undertaken by colleagues from the Judith Lumley Centre with experience in maternal and child health, resulting in minor changes to formatting. The focus group schedule was also piloted by colleagues with experience in maternal and child health. As a result, a question was added to explore whether the role of SILC-MCHN changed over the trial period.

Written consent was obtained from the SILC-MCHNs prior to the focus group, which was audio-recorded and transcribed verbatim. Return of the survey was taken as consent for that component.

### Exploring the views of MCH coordinators: Semi-structured interviews

The views and experiences of MCH coordinators who were involved in establishing the drop-in centres were explored via semi-structured face-to-face interviews. The interviews were conducted at a location and time convenient to the MCH coordinators between March and May 2013. The interviews explored the implementation of the SILC interventions within the universal MCH service, the perceived benefits of the interventions, the challenges faced during the trial period, and any other issues the MCH coordinator wished to discuss. Following written consent, the MCH coordinator interviews were audio-recorded.

### Data management and analysis

All sources of data were de-identified. Unique identifying numbers were allocated to LGAs and pseudonyms assigned to individuals. The three LGAs are referred to as A, B and C in this paper.

Audio files from the SILC-MCHNs’ focus group and the MCH coordinator interviews were transcribed verbatim. Open-ended responses from the SILC-MCHNs’ survey were entered into an Access database verbatim. Inductive thematic analysis was undertaken: first, data immersion was undertaken, where the transcripts were read and re-read to gain an understanding of the data, then data were coded. ‘Basic themes’ with similar responses were grouped together into ‘organisational’ themes [41, 42]. From these groups, key ‘global’ themes were identified [41, 42]. Analysis was undertaken independently by RLC and HLM then compared to ensure validity of emergent themes, with any differences in interpretation of data discussed and resolved.

Quantitative data from the SILC-MCHN survey were entered onto an Access database and analysed using Stata version 11 [43]. The SILC-MCHNs’ diaries and visitors’ comment books were poorly completed. From 326 drop-in sessions, there were 54 SILC-MCHN diary entries completed, of which 47 were completed by one SILC-MCHN. There were 22 entries in the visitors’ comments books in total. As a result, these data were not analysed further, and are not included in this paper.

## Results

### Description of drop-in centres

**Fig. 1:**
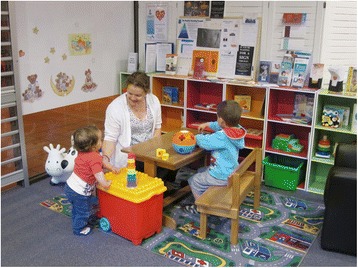
Local Government Area A: Parenting drop-in centre

LGA A is located in regional Victoria, with 1300 births registered in the LGA per year [44]. At the time the SILC study commenced, the LGA had been awarded government funding to establish a service aimed at parenting support more broadly, not solely focussed on breastfeeding, and a local decision was made to combine this funding with the allocated SILC trial funds and establish a parenting drop-in centre (Fig. 1). The drop-in centre was set up in a local shopping mall and was open weekdays between 10 am and 2 pm. It was primarily run by volunteers, who received training in peer counselling. The SILC trial funding allowed a SILC-MCHN to be present between 11 am and 1 pm daily to address any issues that required specialist lactation consultant input. The drop-in centre was promoted within the local hospitals, with flyers distributed by midwives in the maternity units, neonatal special care units and domiciliary services. These flyers were also available in the maternal and child health centres and a Facebook page was created to promote the service. LGA A was the only LGA able to recruit and maintain volunteer staff.

**Fig. 2:**
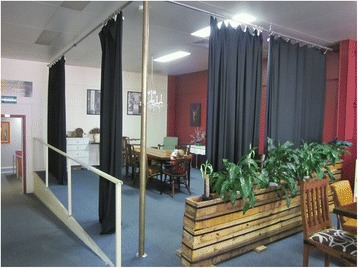
Local Government Area B: Drop-in centre in local café in town 2

**Fig. 3:**
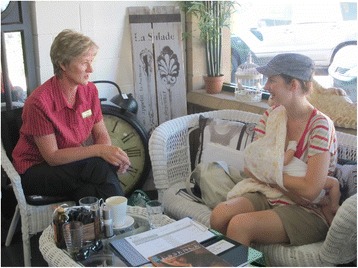
Local Government Area B: Drop-in centre in local café in town 1

LGA B is located in rural Victoria and has approximately 560 births per year registered in the LGA [44]. One SILC-MCHN was employed to run the drop-in sessions for two to three hours, three times per week. Three different locations were used for this drop-in service, in different country towns within the LGA – two in local cafes, one in a local MCH centre. The two cafes set up an area for breastfeeding mothers to gather and discuss their breastfeeding experiences and any concerns or issues they were having. Posters were displayed during these sessions to make the drop-in sessions visible to the public, and breastfeeding information was available. One of the cafes had the ability to close off a private area for breastfeeding as needed (Fig. 2). The other cafe location did not have this option. The area allocated for this drop-in centre was in the front window of the cafe, which faced out onto the street (Fig. 3). Promotion of the drop-in centres was via flyers in MCH centres, general practice clinics, hospitals, kindergartens and child care centres. There was an article in the local newspaper and the drop-in service was advertised on local radio. Information was also provided on the LGA website. LGA B attempted to recruit volunteers through existing community breastfeeding support agencies, such as the Australian Breastfeeding Association, but this was unsuccessful.

**Fig. 4:**
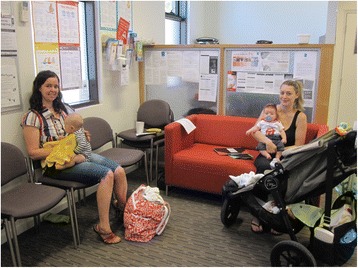
Local Government Area C: Drop-in centre in Maternal and Child Health centre

LGA C was the largest LGA in this trial arm, located in metropolitan Melbourne, with approximately 2700 births per year registered in the LGA [44]. The drop-in centres were located in MCH centres (Fig. 4) in three different suburbs within the LGA, to increase the access across the large geographical area the LGA covered. Sessions lasting three and a half to five hours per week were held three times per week, one at each location. To promote the drop-in sessions, flyers were distributed by the LGA MCHNs during the first Key Ages and Stages home visit. An article was also printed in the local newspaper. Social media (predominantly Facebook) was used by local mothers to promote the drop-in centres. LGA C also attempted to recruit volunteers through existing community breastfeeding support agencies but they too were unsuccessful.

### Drop-in centre attendance

Table 2 describes drop-in centre activity in each LGA including the number of drop-in sessions, and the number of attendances for breastfeeding support and/or information in each LGA during the trial period. Because women could attend the drop-in centres as often as they wished, the number of attendances includes women who visited on multiple occasions. Overall, the drop-in centres were not well attended; across the LGAs, there was an average of one attendance per session for breastfeeding support/information.

**Table 2:** Drop-in sessions and attendances for breastfeeding support at SILC drop-in centres in LGAs allocated to home visit plus drop-in centre trial arm^a^

Location	LGA A Regional	LGA B Rural	LGA C Metropolitan	Total
Hours open	4 h/day 5 days/week	2–3 h/day 3 days/week	4–5 h/day 3 days/week	
Total sessions open	140	92	87	319
Number of volunteers, mean (range)	2 (0, 6)	–	–	–
Attendances for breastfeeding support/information				
Attendances for breastfeeding support/information, n (%)	148 (15)	78 (83)	229 (84)	455
Average attendances/session	1	0.8	2.6	1.4
Infant age (weeks):mean (SD) and median (range)	17 (17.0) 11 (0, 100)	8 (10.3) 4 (0, 60)	5 (4.7) 4 (0, 26)	9 (12.1) 5 (0, 100)
First baby, n (%)	78 (53)	49 (63)	153 (67)	280 (62)
First visit, n (%)	99 (66)	52 (67)	158 (69)	307 (67)

^a^Includes SILC trial run in period

*SILC* Supporting breastfeeding In Local Communities, *LGA* Local Government Area

The median age of infants when women attended the drop-in centres for support with a breastfeeding issue was 11 weeks in LGA A, four weeks in LGA B, and five weeks in LGA C. Over half of the mothers attending were first time mothers across all LGAs.

Reasons women attended the drop-in centres are outlined in Table 3. Seeking breastfeeding support and/or having breastfeeding issues were reported as reasons for drop-in centre visits in the majority of attendances in LGA B and LGA C. However, only 15% of attendees in LGA A (the parenting centre) reported these as the reason for attendance. Other reasons included to meet or help other mothers, to feed their baby, or for parenting information.

**Table 3:** Women’s reasons for attending drop-in centres (data from drop-in centre log books)^a^

	LGA A *n* = 1014		LGA B *n* = 94		LGA C *n* = 274		Total *n* = 1382	
	n	%	n	%	n	%	n	%
Breastfeeding reasons	148	15	78	83	229	84	455	33
Breastfeeding issue	106	10	38	40	162	59	306	22
Breastfeeding support	126	12	61	65	81	30	268	19
Social support								
Meet other mothers	31	3	9	10	1	<1	41	3
Help other mothers	19	2	7	7	1	<1	27	2
Parenting								
Feed my baby^b^	412	41	5	5	14	5	431	31
Parenting information^d^	129	13	–	–	–	–	–	–
Other^c^	590	58	9	10	26	9	625	45

^a^Participants could indicate multiple options, so % could add to >100

^b^Included both breastfeeding and formula feeding

^c^Reasons included using the facilities, weighing baby and having a rest

^d^Only an available option for LGA A

*LGA* Local Government Area

### SILC-MCHNs’ and MCH coordinators’ views and experiences of the drop-in centres

Seven SILC-MCHNs were employed to run the drop-in centres and all completed the written survey; six following the SILC-MCHN workshop held during the last weeks of the trial period and one via email, as she was unable to attend the workshop. The majority were in the age group between 45 years and 64 years (*n* = 6). Six of the seven SILC-MCHNs were registered midwives and MCH nurses (one SILC-MCHN no longer held her midwifery registration but was a registered MCH nurse); four were currently certified International Board Certified Lactation Consultants (IBCLCs); and one SILC-MCHN was a previously certified IBCLC. On average the SILC-MCHNs had 17 years’ experience in midwifery (ranging from 3 years to 35 years); 12 years’ experience in MCH (ranging from 3 to 30 years); and, for those with relevant IBCLC qualifications, 14 years’ experience as IBCLCs (ranging from 1 to 27 years).

Six SILC-MCHNs also participated in the focus group. Four MCH coordinators and managers from the three home visit plus drop-in centre LGAs were identified and invited to participate in the interviews; all consented and were interviewed between March and May 2013. Data from all sources have been combined.

Overall, the SILC-MCHNs and MCH coordinators described many factors that they believed had influenced the success (or not) of the drop-in centres. The practicality of setting up the drop-in centres in accessible locations and encouraging women to attend was complex. Thematic analysis identified two global themes: *implementation challenges* and *the work of SILC-MCHNs*. Within each global theme, three organising themes were identified. Those relating to implementation of drop-in centres were: *accessible, available space; recruiting volunteers to provide peer support*; and *frustration when women did not attend*. Those relating to the work of SILC-MCHN were: *satisfying and rewarding work; juggling roles*; and *benefits to women and babies*.

#### Implementation challenges

Implementing the drop-in centres proved challenging for the participating LGAs. In particular, identifying an accessible and available space, recruiting volunteers to provide peer support, and frustration when women did not attend were obstacles encountered by SILC-MCHNs and MCH coordinators when establishing the drop-in centres.

### Accessible available space

Issues with drop-in centre location were raised by the SILC-MCHNs and MCH coordinators. This included the challenge of finding a suitable location. Two LGAs moved the location of their drop-in centre during the trial period; one moved due to poor attendance, the other changed the locations to be in a more public space.

“*There were some logistical difficulties like finding a drop-in [space], finding a suitable building, arranging times.”* (Olivia, MCH coordinator)

*“We had one originally . . . and our numbers were pretty poor . . . so then we moved it . . . [near public transport, and now] it’s near a shopping centre where people are and the numbers were much better there.”* (Naomi, MCH coordinator)

*“We started off with [drop-in centres] at some of the [MCH] centres but we quickly moved them into cafes because we thought that was sort of a more interesting way to have a go at it . . . ”* (Quinn, MCH coordinator)

### Recruiting volunteers to provide peer support

Another challenge highlighted in the SILC-MCHN focus groups and in the MCH coordinator interviews was the difficulty recruiting and maintaining a volunteer workforce to offer peer support in the drop-in centres. Only one LGA was able to recruit and maintain volunteer staff.

*“Obtaining and retaining volunteers is really difficult.”* (Kate, focus group)

*“We just got nowhere . . . we tried . . . we just couldn’t get the volunteers. I don’t know why. Maybe we didn’t market it properly. [It] would have been great to have that second person.”* (Naomi, MCH coordinator)

### Frustration when women did not attend

Most of the SILC-MCHNs reported feelings of frustration, running drop-in centres with low attendance rates, and many had sessions where no women attended. The SILC-MCHNs felt their time might have been better used in other activities but they were obliged to remain at the drop-in centre.

*“At times frustrated due to lack of clients / mothers. Very satisfied with outcomes when [mothers] did attend.”* (Tracy, SILC-MCHN survey)

*“Very satisfied when women attended but found the drop-in sessions a bit frustrating when no one attended and you knew that you could be utilising that time doing home visits.”* (Amy, SILC-MCHN survey)

One of the MCH coordinators acknowledged the difficulties of low attendance, but described this as one of the challenges faced by many services in smaller, rural areas. She did not consider that this diminished the value of the service to the women who did access the drop-in centres.

*“It’s the same with offering any service in country areas, population density is a real issue in terms of cost because you might run a group in Melbourne and have eight or ten participants, but [country towns] only have two, but it’s still as valuable to those two.”* (Quinn, MCH coordinator)

During the focus group discussion, the SILC-MCHNs who ran the drop-in centres offered several theories about why the drop-in centre attendances were lower than anticipated. Some believed that the women found it difficult to travel within the first few weeks and may have thought it was too difficult to go out with their new baby to seek breastfeeding support.

*"People didn’t want to travel with a 7 day old baby . . . crying and screaming all day and you’re tired and you don’t want to go out*." (Georgia, focus group)

Another reason SILC-MCHNs felt women may not have attended was a lack of privacy, particularly where the centres were established in a public space, which may have been confronting for new mothers seeking breastfeeding help and support. With the drop-in centre set up in the front window of a local café (as was the case in one LGA), the SILC-MCHNs considered that breastfeeding mothers may have felt very exposed, and consequently, they did not utilise the service. (The use of a cloth over the baby and the mother’s breast in Fig. 3 is an example of a mother’s attempt for privacy in this setting.) This was also noted by an MCH coordinator, who felt a cultural shift was needed before women could fully embrace breastfeeding in public places in their small community.

“*We ran [drop-in centres] in cafes and that really wasn’t successful. I suspect that’s quite a cultural change to be part of.*” (Quinn, MCH coordinator)

One LGA reported having a highly culturally and linguistically diverse population, which may have impacted on women’s capacity to get out of the house to seek breastfeeding support.

*“I think [part of the LGA] has a lot of non-English speaking [women] and they have a lot of cultural limitations as well. Not allowed to leave [the house] for 40 days and all that sort of stuff. Or no transportation because the husband’s got the car and they can’t get out . . . They’re often isolated because their family members are [overseas] so they don’t know how to . . . they’re too scared to get out and mix too much.”* (Georgia, focus group)

The SILC-MCHNs in the focus groups reported encouraging women to come to drop-in centres for reasons other than breastfeeding help and support, in order to get the women to attend. A SILC-MCHN from one LGA reported encouraging women to use the SILC drop-in centre as a ‘test run’ for taking a newborn out in public. Another would encourage women to bring their newborn in to be weighed, as they felt women were concerned with infant weight and more likely to attend the drop-in centres if their baby would be weighed.

*“[Women] would say “oh I’m not ready to go out [with my baby]” and I would say “well this is a really safe place with a spot there. How about using it as your trial run for going out for the first time?””* (Kate, focus group)

*“You’d say “come back and get them weighed” rather than [focusing on] feeding ... because they were really worried about the weight they would come to the drop in [centre].”* (Georgia, focus group)

### The work of SILC-MCHNs

SILC-MCHNs reported three main factors in relation to their work during the trial period: satisfying and rewarding work; juggling roles; and benefits to women, babies and the community. The SILC-MCHNs enjoyed the work they were doing to support breastfeeding mothers and their families, but some also encountered difficulties managing workloads for their different roles.

### Satisfying and rewarding work

A strong theme that came through from the focus group discussion was the satisfaction the SILC-MCHNs felt in their work. They reported feeling satisfied when they were able to help a mother feed her baby or resolve other early parenting issues, although no specific examples were mentioned.

*“[I] really enjoyed the role and would love to see it carried on.”* (Tracy, SILC-MCHN survey)

*“[I] felt I did make a difference to how women felt about themselves and their baby - hopefully I helped to empower those few women I saw to try new ways of thinking about their feeding.”* (Whitney, SILC-MCHN survey)

### Juggling roles

Six of the seven SILC-MCHNs involved in running the drop-in centres were also responsible for the provision of home visits as part of the SILC trial. This presented challenges in ensuring the SILC-MCHN could physically get to the drop-in centre in time to run the sessions. This was particularly difficult in the LGAs with larger geographical areas to cover and resulted in the SILC-MCHNs having to undertake complex planning around their schedules.

*“Extremely challenging, depending geographically where you were going for the next visit . . . You had to be very organised and pre-plan the day before. I always knew geographically exactly where I was going the next day and had it worked out to the minute, pretty well.”* (Kate, focus group)

*“[I] very often ate lunch in car on the way to the drop-in session . . . because if I’m travelling from one end of the shire to the other it’s an hour driving so I could have a home visit in [one town] and then have to be in [another town] for the drop-in sessions so that was quite challenging.”* (Frances, focus group)

This challenge was acknowledged by the MCH coordinators, who were responsible for managing the SILC-MCHN and the universal MCH services.

*“It was difficult to schedule a staff member who’s doing both home visiting and staffing a drop-in . . . It made her day a bit tight sometimes.”* (Olivia, MCH coordinator)

For example, one LGA introduced a strategy to alleviate the issue of the SILC-MCHN juggling roles. The SILC-MCHN ran the drop-in centre in the morning and allocated the afternoon for completing other duties. On Fridays, when the drop-in centre was open for five hours, the SILC-MCHN was rostered to facilitate the drop-in centre only, and was not allocated any additional duties, such as home visits or other routine MCHN workload.

### Benefits to women, babies and the community

All the SILC-MCHNs were positive about the support they were able to provide when women did attend the drop-in centres. They also reported seeing the benefit of two or more mothers attending the drop-in centre at the same time so that they were able to talk to each other and compare experiences, and offer informal peer support.

*“With other mums there the conversation flows. When you’ve got 2 or 3 [mothers] they’re looking at other people that have similar concerns that they have.”* (Isobelle, focus group)

*“I think a lot of what we do is to assist / facilitate women to network.*” (Benita, SILC-MCHN survey)

Another perceived benefit was that the community as a whole were able to be educated about breastfeeding through the availability of the drop-in centres. One LGA drop-in centre was located in a local cafe at lunchtime, so was very visible within the community, and the SILC-MCHN reported that this attracted questions and interest from those coming to the cafe during the busy lunchtime period.

*“I had [the drop-in centre] at a café . . . I was usually up the front in the windows space in the lounge area I did have a lot of people [because] it is a busy cafe, so a lot of people would ask what I was doing there . . . [I] had an elderly gentleman sit with me last week who thought he better move on before they got the wrong idea!”* (Frances, focus group)

## Discussion

The LGAs developed and ran very different models of breastfeeding drop-in centres during the SILC trial. Overall, attendance was low, with an average of only one attendee per session. A range of complex factors impacted on the establishment of the drop-in centres and also the attendance by women. Two global themes were identified: *the implementation challenges*, including themes of accessible, available space, recruiting volunteers to provide peer support, and frustration when women did not attend; and *the work of SILC-MCHNs*, including themes of satisfying and rewarding work; juggling roles; and benefits to women, babies and the community.

Attendance rates varied greatly between and within LGAs. LGA A experienced the greatest demand for drop-in centre services during the trial, though primarily for parenting support, not specifically for breastfeeding. LGA C’s attendance rates varied greatly between their three drop-in centres, highlighting the importance of location and opening hours to the success of the service. A number of other studies of breastfeeding drop-in centres and drop-in centres established for health promotion and social support activities have also reported on these issues [16–18, 24, 26, 29, 30, 45], i.e. the importance of the location being easily accessible [18, 24, 30], and that the hours suit the target population, as opposed to what is convenient for the staff running the service [16, 17, 24, 26, 29].

Another possible reason for the lack of attendance may be that women are increasingly seeking parenting support [46] and, by extension, infant feeding support from social networking sites. They are no longer required to leave their home to access support and advice, which can now be accessed from their smartphone, tablet or computer [46]. As women perceive this online activity as providing valuable support [47, 48], they may be satisfied to engage with their online communities and not feel the need to attend services like drop-in centres. Further investigation of women’s experiences of breastfeeding support during the SILC trial, including the sources of breastfeeding advice and support, will be presented in a future publication [49].

One of the drop-in centres from LGA B was held in the front window of the local cafe during lunchtime. It was located in a small, country town and was very visible. Given some women’s reluctance to breastfeed in public [50–52], it is possible this public location may have made women feel uncomfortable seeking breastfeeding advice and support in such a visible space.

Due to funding-related time constraints, the drop-in centres in this study were open for nine months in terms of trial data collection, with an additional two months for the set up and run-in period. It is possible that there may have been greater attendance if the services had been able to have a longer run-in period, allowing time for them to become more established within the community. Other studies have reported that ongoing adaptability of drop-in centres and the ability to engage and partner with, and adapt to the needs of, the target population may contribute to the overall success of a service [24, 25]. It has also been reported that establishing drop-in centres takes significant time [25, 26, 30, 32]. Since the completion of the SILC trial, two of the three SILC LGAs are continuing to offer breastfeeding drop-in centres, with an increase in attendance numbers (MCH and Immunisation Clinical Coordinator: LGA A, personal communication, 8 November 2016; Team Leader MCH: LGA C, personal communication, 15 November 2016). A representative from one LGA reports the breastfeeding drop-in centres are now considered an integral part of the care provided to women and there are plans to extend the services to four days per week (MCH coordinator: LGA C, personal communication, 8 November 2016).

### Strengths and limitations

This study explored the SILC-MCHNs’ and MCH coordinators’ views and experiences of implementing the SILC drop-in centres. The use of multiple data sources provided an opportunity to explore what was successful and what challenges were faced from each viewpoint, enabling a multi-level evaluation of the services. All MCH coordinators from the drop-in centre LGAs were interviewed and all SILC-MCHNs employed on this trial completed the written survey to give their experiences and feedback. All but one SILC-MCHN also participated in the focus group discussion. The high participation rate in key evaluation activities allows a complete picture of the intervention to be ascertained and strengthens the validity of the results of this evaluation.

The SILC-MCHNs employed in SILC were self-selected, motivated and dedicated to promoting and supporting breastfeeding for their communities, and all had undertaken additional training and breastfeeding education. These results reflect the SILC-MCHN views and experiences and may not be generalisable to all MCHNs, as there are likely to be differing views about the importance of breastfeeding and how best to support new mothers with infant feeding.

## Conclusion

This study explored the implementation of community-based breastfeeding drop-in centres and evaluated the service from the views and experiences of the SILC-MCHNs employed to run them, and from the MCH coordinators tasked with overseeing the drop-in centres from a management perspective. Overall, the SILC-MCHNs enjoyed the work they were doing supporting breastfeeding and felt they could make a difference for women and families, but attendance was low, and getting women to attend proved difficult. It is possible that running the drop-in centres for a longer period of time may have improved attendance rates, as the centres became more integrated into, and accepted by, the community. Further research is needed to determine if community-based drop-in centres are effective in supporting women to breastfeed in populations where breastfeeding rates are low.
